# Effects of Dust Addition on the Reactivity and High-Temperature Compressive Strength of Ferro-Coke

**DOI:** 10.3390/ma18245637

**Published:** 2025-12-15

**Authors:** Rongrong Wang, Siqi Li, Yongsheng Yang, Runsheng Xu, Jianliang Zhang, Yu Zeng, Yuchen Zhang, Bin Wu

**Affiliations:** 1 State Key Laboratory of Advanced Metallurgy, University of Science and Technology Beijing, Beijing 100083, China; rongrong.wang@ustb.edu.cn (R.W.);; 2 Technical Support Center for Prevention and Control of Disastrous Accidents in Metal Smelting, University of Science and Technology Beijing, Beijing 100083, China; 3 School of Metallurgical and Ecological Engineering, University of Science and Technology Beijing, Beijing 100083, China; 4 Angang Steel Company Limited, Anshan 114009, China

**Keywords:** iron-carbon-containing dust (ICD), ferro-coke, reactivity, high-temperature compressive strength, pore structure

## Abstract

This study evaluated how raw-material compounding strategies affect the high-temperature compressive strength of ferro-coke. Ferro-coke was prepared using varying additions of iron-carbon-containing dust (ICD) and different gasification temperatures. High-temperature experiments were conducted to study the reactivity (CRI) and hot strength of ferro-coke, and fracture morphologies were examined by microscopy and Micro-CT. The results show that both the increase in ICD addition amount and gasification temperature would increase the reactivity and decrease the high temperature of ferro-coke. As ICD addition increased from 5% to 20%, the reactivity of the ferro-coke rose from 58.70% to 76.32%, and high-temperature strength decreased from 3260 N to 1954 N at 800 °C. This trend is attributed to catalytic components in ICD (e.g., Fe, Zn), which would accelerate gasification, increase porosity, and reduce high-temperature strength. Similarly, increasing the gasification temperature from 1000 °C to 1200 °C enhanced reactivity from 51.95% to 65.51%, but the hot strength was reduced. The higher carbon gasification rate at elevated temperature increases porosity and weakens the coke matrix, lowering compressive strength.

## 1. Introduction

Low-carbon blast furnace ironmaking is critical for energy conservation, emission reduction, and sustainable development in the steel industry [1,2]. The carbon-reduction potential achievable through conventional burden preparation, operation, and management is nearing its limit; deep decarbonization therefore requires the precise design of novel burden materials and smelting schemes tailored to the functional roles of raw materials in a blast furnace [3,4]. Ferro-coke, as a highly reactive coke that can comprehensively utilize iron-bearing and coal-based resources while effectively enhancing the energy-saving and emission reduction potential of blast furnaces, is currently a key focus of research and development. Metallurgical dust and sludge are major solid by-products of the steel industry, output of which typically accounts for 8–12% of crude steel production. Given the high Fe and C contents, dust and sludge are suitable feedstock for ferro-coke, which would contribute to energy-saving and emission reduction targets [5,6]. Ferro-coke that couples iron-bearing and coal-based resources and exhibits high reactivity, is thus a promising route to improve blast furnace efficiency and lower emissions [7].

For the application of ferro-coke that contains iron ore fines in a blast furnace, laboratory trials, pilot-scale trials, and industrial trials have all been conducted and reported. Wang et al. [8,9] hot-pressed Shenfu coal with iron ore fines and asphalt binder followed by carbonization, producing ferro-coke with an initial reaction temperature of 665.3 °C and an average cold compressive strength of 5.89 MPa. Chu et al. [10,11] proposed a shaft furnace process for the production of ferro-coke. By hot-pressing the mixture of anthracite, bituminous coal, and iron ore fines at 300 °C and carbonizing the briquettes at 1000 °C for 4 h, ferro-coke with compressive strength of 5049 N, CRI of 61.08%, and CSR of 51.23% could be produced. A cold-press route was further developed to mitigate environmental and safety issues during hot pressing. Bi et al. [12] added iron ore fines to a standard coking coal blend (fat coal, coking coal, gas-fat coal, lean coal) and produced ferro-coke that has a CRI of 64.07% and a CSR of 21.63% by using a 5 kg pilot coke oven. Ren et al. [13] used a 40 kg oven to produce ferro-coke by using a blend of 10% iron ore fines and 90% one-third coking coal; the product has a CRI of 42.5%. Zhang et al. [14,15] employed a 2 kg oven to study the effects of different iron oxides on coking behavior and properties of ferro-coke. Japan has led ferro-coke research and scale-up. Nippon Steel produced ferro-coke in a conventional coke oven and applied it in a blast furnace; industrial trials reduced the thermal reserve zone temperature by 186 °C and increased furnace efficiency by 6.8% [16]. JFE Steel used a shaft furnace to produce 2500 t of ferro-coke, which was then charged into a 5153 m^3^ blast furnace, achieving stable operation and significant fuel savings. The above results validated the feasibility of using ferro-coke in blast furnace applications, while the investigation and application of dust-rich ferro-coke remain relatively uncommon [17].

In the previous work by authors’ research group [18,19,20,21], ferro-coke that contains metallurgical dust and weakly caking coal was prepared in laboratory. The strength of ferro-coke after reaction and volume evolution during the gasification process were investigated, while the change patterns of the ferro-coke’s strength during gasification and the associated fragmentation mechanisms have not yet been studied. For coke used in a blast furnace, extensive research has linked coke strength and fragmentation to intrinsic factors (pore architecture, mineral matter, internal cracks) and extrinsic factors (mechanical loads, thermal stresses, and gas–solid reactions), which provide a foundation for the further study of dust containing ferro-coke’s strength.

Therefore, this study aims to investigate the variation in high-temperature compressive strength of dust-containing ferro-coke under the different blending strategies of ICD and weakly caking coal (e.g., blending ratios and briquetting parameters). The objectives are to identify the determinants of ferro-coke’s high-temperature fragmentation and to further reveal the underlying response mechanisms triggered by gasification. These findings will provide a theoretical basis for suppressing the pulverization of dust-containing ferro-coke and enhancing its utilization rate within the blast furnace charge layer, thereby guiding the targeted regulation of ferro-coke strength for blast furnace applications.

## 2. Materials and Methods

### 2.1. Raw Materials

The coal powder (RM for short) and binder coal tar pitch (LQ for short) were sourced from a Chinese steel plant. The iron-carbon-containing dust (ICD for short) was collected from the dedusting system of the same plant.

As shown in Table 1, RM coal has a fixed carbon content of 66.30%, an ash content of 12.13%, and a volatile matter content of 21.57%. Ultimate analysis conducted in accordance with GB/T 31391-2015 [22] “Elemental analysis of coal” using an elemental analyzer (Vario MACRO cube, Germany) shows that the C, H, N, S, and O element content of RM is 77.14%, 4.28%, 2.48%, 1.328%, and 2.74%, respectively. Composition of ash was determined by X-ray fluorescence (XRF), and the results are listed in Table 2.

**Figure 1:**
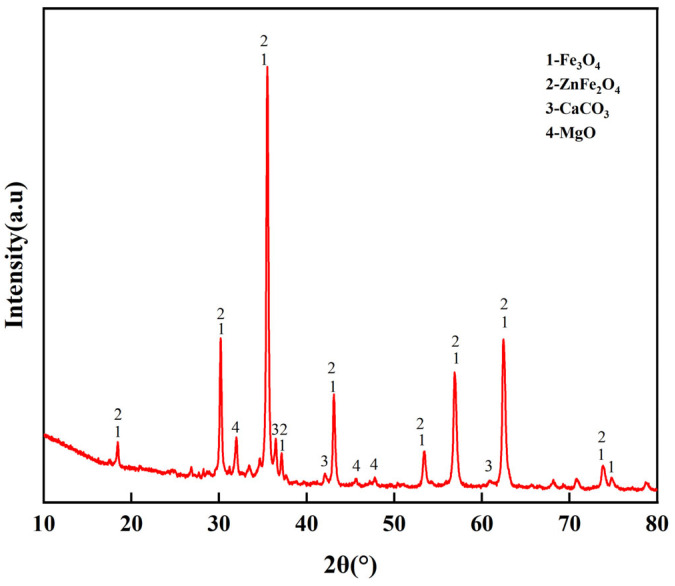
XRD of ICD.

As indicated by Table 3 and the XRD pattern of ICD (Figure 1), Fe occurs as iron oxides with mixed valence (magnetite, Fe_3_O_4_ and wüstite, FeO), and as zinc ferrites formed by reactions between ZnO and iron oxides (e.g., ZnFe_2_O_4_, ZnO·Fe_2_O_3_), which are the principal Zn carriers. Since ferro-coke is produced under high temperatures, basic and acidic oxides in the dust–coal matrix can form complex silicates (e.g., 2CaO·SiO_2_).

The compositions of refining dust and EAF dust were analyzed using XRD and XRF, with the results presented in Table 3 and Figure 1. The EAF dust is primarily composed of iron, whereas the refining dust contains relatively higher proportions of Ca and Mg, but low total iron (TFe) content. In the EAF dust, iron mainly exists in the forms of iron oxides (Fe_3_O_4_, FeO) and zinc ferrite spinel (ZnFe_2_O_4_). The refining dust is predominantly composed of MgO, Ca(OH)_2_, CaCO_3_, Ca_3_SiO_5_, and Fe_3_O_4_.

The data in the table represent the main chemical composition analysis results of the iron-carbon-containing dust (ICD) (expressed in oxide forms). The balance consists primarily of carbon, volatile matter, and trace elements.

### 2.2. Experimental Methods

#### 2.2.1. Sample Preparation

RM coal powder was first crushed to a particle size below 0.074 mm using a laboratory roll crusher. It was then mixed with coal tar pitch and ICD according to predetermined mass ratios. The resulting mixture was pressed into cylindrical briquettes (abbreviated as CDB) using a 25 mm diameter die at a pressure of 30 MPa, with a holding time of 30 s. Ferro-coke was subsequently produced through further carbonization of the CDB.

#### 2.2.2. Carbonization and Gasification Tests

**Figure 2:**
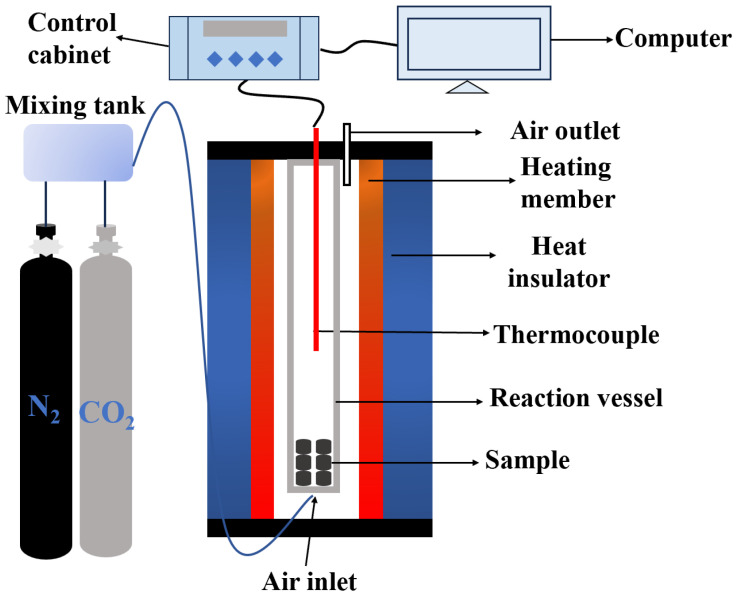
Schematic diagram of the carbonization and gasification furnace.

Carbonization of CDB and gasification of ferro-coke were performed on an SYD-224I dual furnace, the structure of which is shown in Figure 2.

The carbonization process of CDB: A CDB was placed in a graphite crucible (outer diameter 30 mm, inner diameter 27 mm, height 50 mm). A cylindrical iron weight (25 mm × 25 mm) was placed on top to apply a uniform dead load. The crucible was inserted into the reaction tube and heated at 10 °C/min to the target temperature, then held for 20 min. High-purity N2 (2 L/min) was purged throughout to ensure an inert atmosphere. During this process, the CDB was carbonized in the crucible and produced ferro-coke.

The gasification process of ferro-coke: The furnace was heated at 10 °C/min under N2 (2 L/min) to the set temperature, then switched to CO2 at the same temperature for 2 h. The ferro-coke (carbonized CDB) was put into the furnace for gasification. After gasification, heating stopped and N2 purging (2 L/min) resumed for cooling.

Two factors that influence ferro-cokes’ high-temperature strength were investigated: ICD addition amount and gasification temperature. The experimental conditions are shown in Table 4. To assess the effect of ICD addition amount, ferro-coke containing different mass fractions of ICD (5%, 10%, 15%, 20%) were gasified under identical temperature (1100 °C), then high-temperature strength was measured at specified test temperatures to determine the relationship between ICD addition and maximum hot strength (800 °C, 900 °C, 1000 °C, 1100 °C). To assess the effect of gasification temperature, ferro-coke with the same ICD addition (10%) was gasified at different temperatures (25 °C, 1000 °C, 1100 °C, 1200 °C), then high-temperature strength was measured at specified test temperatures.

#### 2.2.3. High-Temperature Strength Test

**Figure 3:**
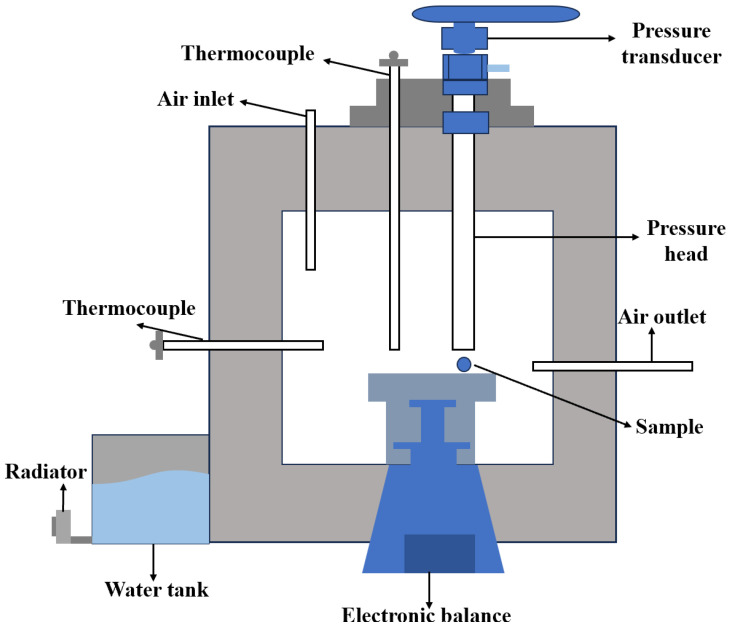
Schematic diagram of high-temperature strength test device.

The maximum high-temperature compressive strength of ferro-coke after gasification was measured using a self-developed high-temperature strength test device. Cylindrical ferro-coke specimens were oriented horizontally, and load was applied laterally via an alumina indenter while an industrial camera monitored fracture (Figure 3). The peak load when the ferro-coke was crushed was recorded.

**Figure 4:**
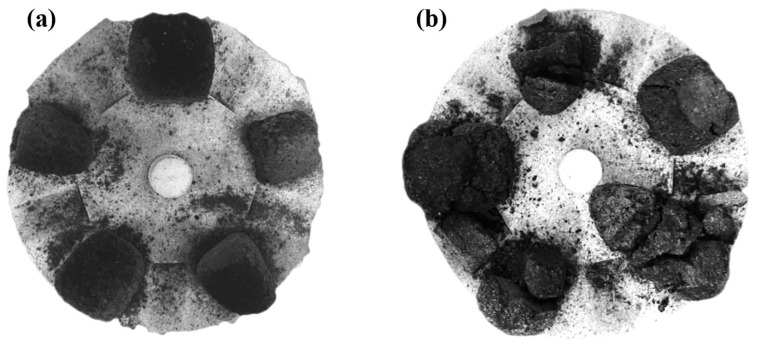
Ferro-coke (**a**) before hot-temperature strength test; (**b**) after hot-temperature strength test.

Place up to five samples onto the rotating sample carrier (Figure 4a), then insert the carrier into the test device and align the recess preceding the first sample with the viewing port; after that, set the heating profile and protective gas flow rate in the control software. Preheat the device to the target temperature under the protection of cooling water and N_2_, then rotate the carrier to position the first sample in the viewing window. Apply a continuous load (e.g., ~4 N to ~20 N) to the sample upon audible fracture and visible cracking, record the maximum load, and repeat the test procedure for all samples. Cool down the test device under N_2_ purging until the furnace cools to 300 °C. Take out the samples when they are cooled to room temperature (Figure 4b). Each test in Table 4 was carried out four times to ensure reproducibility, and the results are reported as mean ± standard deviation.

#### 2.2.4. 3D CT Analysis

In order to more accurately and comprehensively explore the internal microstructural changes of the ferro-coke during the gasification process, X-ray micro-computed tomography (micro-CT) was used to analyze the distribution of pores and minerals at different positions within the sample. In this study, ferro-coke samples with varying dust addition levels were scanned using a Micro-CT system (FF35 CT, YXLON, Hamburg, Germany) with a slice interval of 15 μm. The acquired image stacks were subsequently reconstructed into three-dimensional models using Avizo software version 2022.1 for further analysis.

#### 2.2.5. Ultra-Precise 3D Microscopy

In this study, a 3D digital microscope with extended depth of field (Model: VHX-6000, Keyence Corporation, Osaka, Japan) was used to characterize the surface morphology and conduct 3D analysis of the cross-section of ferro-coke after thermal cracking. The sample was securely mounted on the stage, and images were captured using a 20× objective under ring light illumination. By setting the software-defined Z-axis scan range (start and end positions), the system automatically acquired a stack of images along sequential focal planes, and an integrated focus-stacking algorithm was applied to generate an all-in-focus 2D image and the corresponding 3D surface model. All dimensional measurements were performed based on the all-in-focus image and the 3D model. Prior to measurements, the system was calibrated using a certified stage micrometer.

## 3. Results and Discussion

### 3.1. Reactivity of Ferro-Coke

#### 3.1.1. Effect of Iron-Carbon Containing Dust Addition

**Figure 5:**
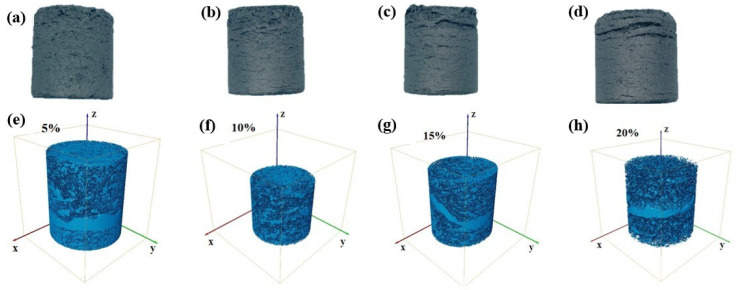
Macroscopic morphologies and pore structure of ferro-coke with different ICD addition amount (**a**) Morphology-5%; (**b**) Morphology-10%; (**c**) Morphology-15%; (**d**) Morphology-20%; (**e**) Pore structure-5%; (**f**) Pore structure-10%; (**g**) Pore structure-15%; (**h**) Pore structure-20%.

**Figure 6:**
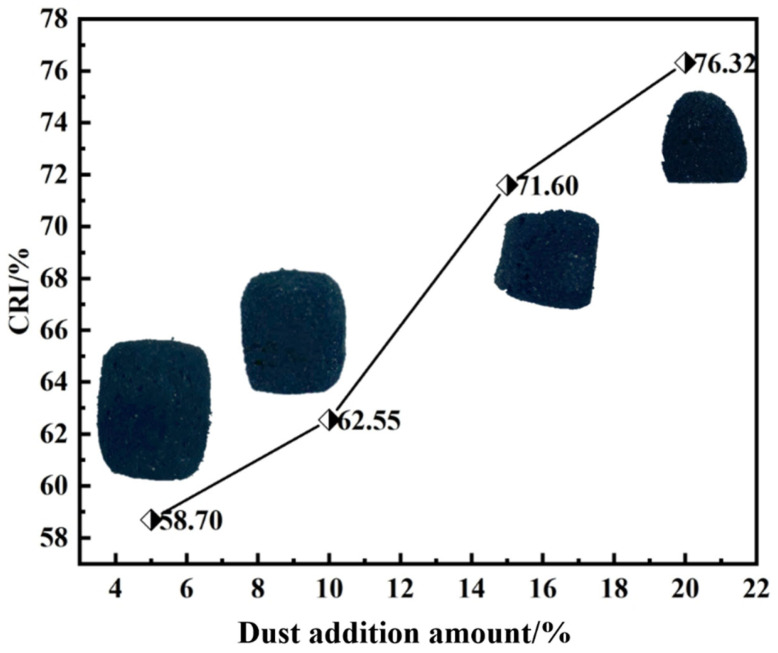
Change in ferro-coke’s reactivity index and post-gasification morphology with ICD addition.

Figure 5 and Figure 6 show the morphology and pore structure of ferro-coke with ICD additions of 5%, 10%, 15%, and 20%. The ferro-coke surfaces display few pores and some top-edge cracks at low addition amount, both pore number and size, as well as crack density increase as the ICD addition increases. As shown in Figure 5, the 3D pore architecture of ferro-coke depends on and correlates positively with the ICD addition. The porosity of ferro-coke is 16.02% at 5% addition, and increases to 27.08% at 10%, 31.20% at 15%, and 33.33% at 20%. The increasing trend of porosity clearly reflects the progressive structural evolution of ferro-coke.

Figure 6 shows the change in ferro-coke’s reactivity index (CRI) and post-gasification morphology with ICD addition. When the ICD addition amount increases from 5% to 10%, the CRI of ferro-coke rises from 58.7% to 62.6%, indicating a modest enhancement. A further increase to 15% and 20% raises the CRI to 71.6% and 76.3%, maintaining the upward trend. The post-gasification morphologies of ferro-coke show a clear volume decrease with higher addition; the result is in consistent with increased reactivity.

According to Figure 1, Fe in the added ICD exists primarily as Fe_3_O_4_ and ZnFe_2_O_4_. A prior work reports [23,24] that zinc acts mainly via ZnO as an oxygen-transfer medium in blast furnace reaction systems. In the oxygen migration framework, ZnO decomposition releases active oxygen that reacts readily with the carbon in ferro-coke and promotes ferro-coke’s gasification. Concurrently, Zn^2+^ formed on carbon surfaces modifies oxygen adsorption, consistent with electron-transfer theory, which lowers the chemisorption barrier for O_2_ on carbon, reduces activation energy, and accelerates the O-C reaction. Zinc also exhibits distinct reductive behavior. At high temperature, ZnO is preferentially reduced by ferro-coke to Zn vapor, which escapes with the gas stream. This alters the internal composition and, via a local volumetric-change effect, increases the porosity of the ferro-coke during the gasification process. The resulting porous structure promotes subsequent gasification by the following: (i) increasing specific surface area and active sites, and (ii) improving permeability and gas diffusion to reaction interfaces, thereby enhancing overall kinetics. Therefore, the addition of ICD in ferro-coke would result in the increase in CRI.

#### 3.1.2. Effect of Gasification Temperature

**Figure 7:**
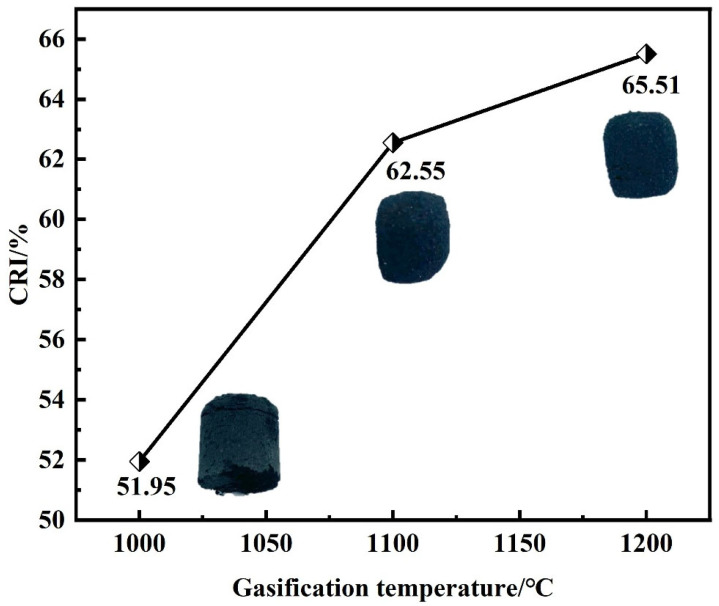
Change in ferro-coke’s reactivity index and post-gasification morphology with gasification temperature.

Figure 7 shows the change in ferro-coke’s reactivity index (CRI) and post-gasification morphology with gasification temperature. When gasification temperature increases from 1000 °C to 1100 °C, CRI rises from 51.95% to 62.55%, indicating a substantial enhancement. A further increase from 1100 °C to 1200 °C raises CRI from 62.55% to 65.51%, which maintains an increasing trend, but the trend tends to level off. High temperature promotes the decomposition of ZnO and accelerates the O-C reaction, thus raising the CRI of ferro-coke. The post-gasification morphologies of ferro-coke show a clear volume decrease with higher gasification temperatures; this result is consistent with increased reactivity.

### 3.2. High-Temperature Compressive Strength of Ferro-Coke

**Figure 8:**
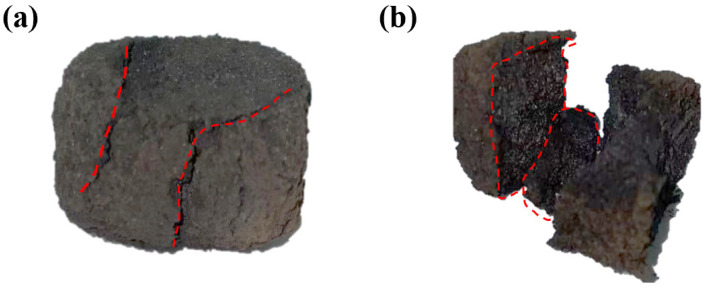
Morphology of ferro-coke sample after hot splitting (**a**) 5%; (**b**) 15%.

Figure 8 shows the cross-section morphologies of ferro-coke after thermal cracking at different ICD additions. At 5% ICD (Figure 8a) addition, the specimen remains largely intact at the peak load, with only a single long crack in the circled region, while at 15% ICD (Figure 8b) addition, the specimen shatters into small fragments at maximum load and loses its original shape. This contrast reflects that ferro-coke has higher compressive strength at lower ICD additions, and so preserves a more integral morphology.

**Figure 9:**
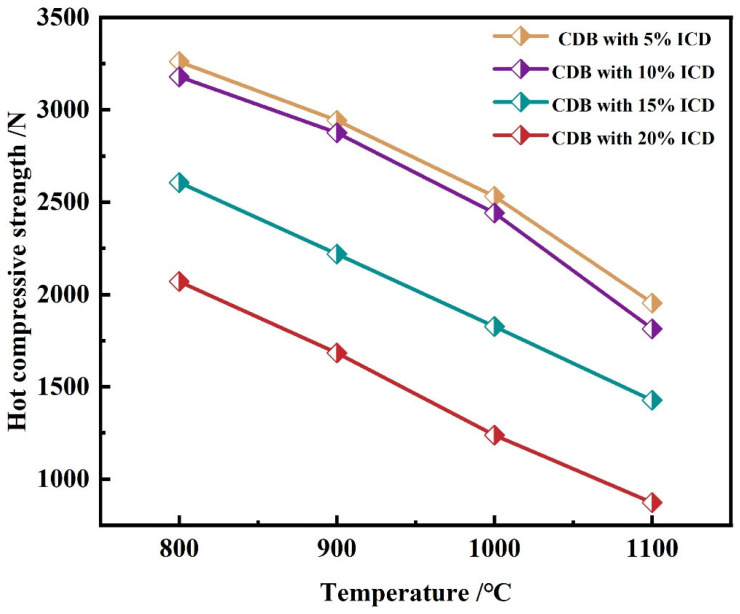
Compressive strength of samples with different ICD addition amounts at different temperatures.

Figure 9 summarizes the maximum high-temperature strength at different test temperatures and ICD additions. The high-temperature strength of ferro-coke consistently declines with increasing ICD addition, which is in line with the change in ferro-coke’s reactivity. At 5% ICD addition, strengths are 3260 N, 2943 N, 2532 N, and 1954 N at the test temperatures of 800 °C, 900 °C, 1000 °C, and 1100 °C, respectively. At 20% ICD addition, the corresponding strength continuously decreases to 2070 N, 1684 N, 1239 N, and 873 N.

According to the plastic coking principle, metallic iron is encapsulated by the liquid caking phase during coke formation and participates in the generation of pore walls. At high temperatures, molten iron interacts with coal-derived binder components and is fixed by the physical encapsulation effect of the coke matrix. Upon cooling, the encapsulated iron migrates toward pore walls, co-forming a composite skeleton with the carbon framework and a hierarchical pore structure. This 3D network of metallic iron and carbon provides mechanical reinforcement to ferro-coke.

During the high-temperature gasification process, ferro-coke undergoes expansion and increased internal porosity. An increased iron oxide content reduces coal expansibility. Once iron oxides exceed a threshold, swelling during the thermoplastic stage is suppressed, and the caking phase cannot fully coat all iron ore particles, leaving some exposed particles at high temperature. These uncoated particles are prone to polymorphic or phase transformations during cooling, disrupting the carbon skeleton. The resulting structural defects lead to lower compressive strength and reduced abrasion resistance in the final ferro-coke. Meanwhile, as ICD content in ferro-coke rises, porosity increases more significantly, as explained in Section 3.1.1, disrupting the 3D network structure of ferro-coke and resulting in lower high-temperature compressive strength.

#### Effect of Gasification Temperature

**Figure 10:**
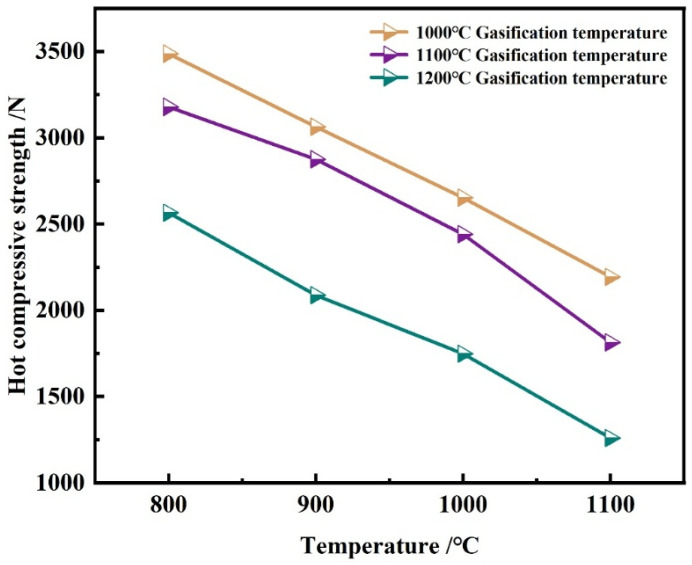
Compressive strength of samples with different gasification temperatures at different temperatures.

Figure 10 reports the maximum high-temperature strength of ferro-coke after gasification at different temperatures. The strength decreases monotonically with increasing gasification temperature, consistent with ferro-coke’s reactivity. When testing the high-temperature strength at 800 °C, for the ferro-coke after gasification under 1000 °C, 1100 °C, and 1200 °C, the strengths are 3487 N, 3179 N, and 2565 N, respectively. The reason can be contributed that, under high-temperature gasification conditions, carbon-based materials in ferro-coke are consumed, leading to the destruction of the network structure and a reduction in the strength. Furthermore, as the gasification temperature increases, the consumption of carbon-based materials rises, causing more severe damage to the 3D network structure and a further decline in high-temperature strength properties.

### 3.3. Microstructure of Ferro-Coke After Gasification and Thermal Cracking

#### 3.3.1. Effect of Iron-Carbon-Containing Dust Addition

**Figure 11:**
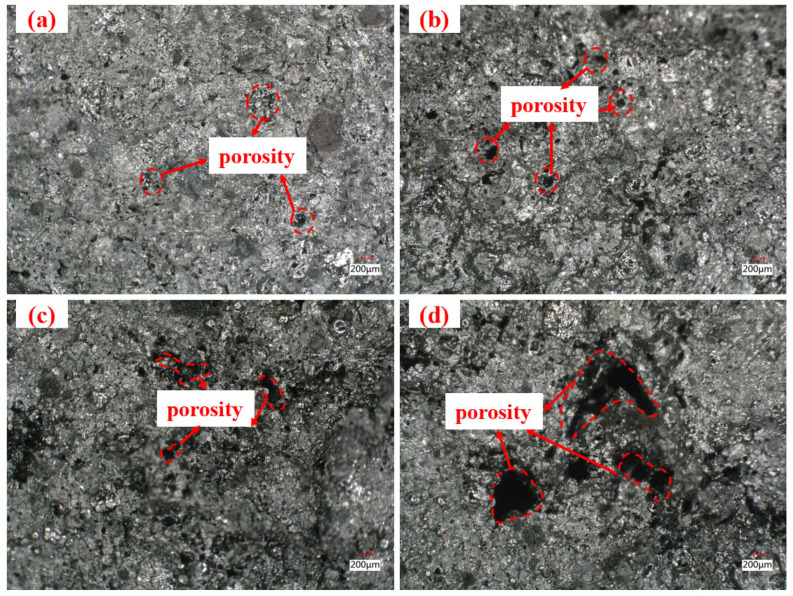
Cross-sectional morphology of ferro-coke samples with different ICD addition amounts (obtained using an ultra-depth microscope): (**a**) 5%; (**b**) 10%; (**c**) 15%; (**d**) 20% (magnification: ×50).

Fracture surfaces of ferro-coke after thermal cracking were examined using a 3D ultra-depth microscope (Figure 11). With increasing ICD addition, sectional porosity increases, small pores are progressively replaced by medium or large pores, and pore walls become thinner. Both the pore number and the size of 5% ICD ferro-coke are clearly lower than that in 20% ICD ferro-coke; this result is consistent with the hot-temperature strength trends in Figure 9.

**Figure 12:**
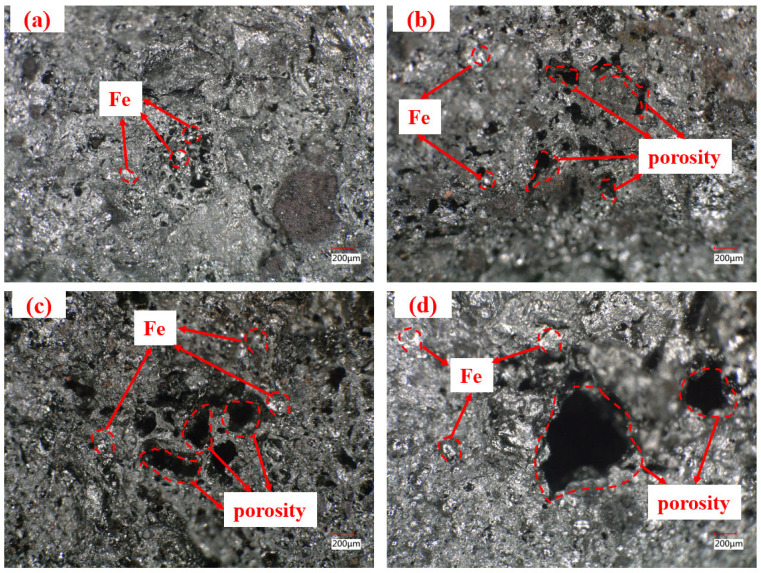
Cross-sectional pore morphology of ferro-coke samples with different ICD addition amounts (obtained using an ultra-depth microscope) (**a**) 5%; (**b**) 10%; (**c**) 15%; (**d**) 20% (magnification ×100.0).

Figure 12 shows a distinct particle interface between ICD and coal particles, with numerous iron-rich pore features distributed around the boundary. This occurs because zinc oxides in ICD are reduced to metallic Zn during carbonization, volatilizes (at about 907 °C), and escapes as vapor from the ferro-coke matrix. By this stage, the coal-derived caking phase has transformed into a carbon matrix and lost its binding ability, so it cannot fill the voids left by evaporated Zn. As dust addition increases, pore density rises and pore size expands. Meanwhile, solid reduction products from the dust form physical barriers in interparticle gaps, hindering effective bonding between coal particles. The poor cohesive properties result in a discrete particle distribution, with structural cracks even forming in certain areas.

#### 3.3.2. Effect of Gasification Temperature

**Figure 13:**
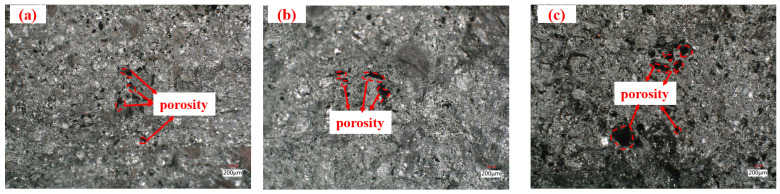
Cross-section of ferro-coke samples at different gasification temperatures (obtained using an ultra-depth microscope) (**a**) 1000 °C; (**b**) 1100 °C; (**c**) 1200 °C (Magnification ×50.0).

Fracture surfaces of ferro-coke samples gasified under different temperatures after thermal cracking were further examined, and the result is shown in Figure 13. With increasing gasification temperature, similar to the effect of ICD addition amount, sectional porosity increases, small pores are progressively replaced by medium and large pores, and pore walls become thinner. In Figure 13a (ferro-coke gasified at 1000 °C), both pore number and size are clearly lower than in Figure 13c (gasified at 1200 °C), which is consistent with the high-temperature strength results in Figure 10.

## 4. Conclusions

This study examines the influence of raw-material compounding on the high-temperature strength of ferro-coke by varying the iron-containing dust (ICD) addition and the gasification temperature, assessing reactivity (CRI) and high-temperature strength, and analyzing fracture morphologies. The main findings are as follows:

(1) Increasing the ICD addition elevates the CRI of ferro-coke. As the ICD addition increases from 5% to 20%, the CRI rises from 58.70% to 76.32%, while the high-temperature strength of ferro-coke after gasification decreases from 3260 N to 1954 N at 800 °C. Iron and zinc in the dust catalyze gasification, intensify internal erosion, and thereby reduce high-temperature strength.
(2) Increasing the gasification temperature also raises CRI. When the gasification temperature increases from 1000 °C to 1200 °C, the CRI increases from 51.95% to 65.51%, accompanied by a decrease in high-temperature strength. Higher gasification temperatures enhance reactivity, increase porosity, and diminish the high-temperature strength.
(3) At 5–10% ICD addition and a gasification temperature of 1100 °C, ferro-coke exhibits comparable strengths, indicating a balance between metallic iron contributing to the load-bearing skeleton and its suppression of coal expansibility. The high-temperature strength reduction in ferro-coke stems from the following two factors: (i) Zn catalyzes carbon gasification; (ii) Zn volatilizes at high temperature, generating additional porosity, enlarging specific surface area, and further accelerating carbon gasification.
(4) Based on the comprehensive analysis of high-temperature compressive strength, reactivity, and structural evolution, an ICD addition of 10 wt.% appears to offer the best balance. Within this range, the beneficial catalytic effect on gasification is present without causing excessive structural degradation or zinc-induced weakening, thereby optimally mitigating high-temperature fragmentation for blast furnace application.

## Figures and Tables

**Table 1:** Proximate analysis and elemental analysis of SM (Air-dried basis; wt.%).

	Proximate Analysis			Ultimate Analysis				
	Fc/%	Ash/%	Vad/%	C	H	N	S	O^a^
RM	21.57	12.13	66.30	77.14	4.28	2.48	1.328	2.74

O^a^ By different (O% = 100%-Ash%-C%-H%-N%-S%).

**Table 2:** Composition analysis of coal ash (wt %).

	SiO_2_	Al_2_O_3_	Fe_2_O_3_	CaO	MgO	TiO_2_	K_2_O	Na_2_O	P_2_O_5_
RM	2.906	35.822	11.321	2.858	0.319	2.832	0.755	0.344	1.043

**Table 3:** Chemical composition of iron-carbon-containing dust (ICD) (wt.%).

TFe	CaO	SiO_2_	MgO	Al_2_O_3_	K	Na	ZnO
32.30	8.06	8.87	4.4	1.32	1.56	1.27	12.46

**Table 4:** Experimental conditions for the high-temperature compressive strength tests of ferro-coke samples.

Addition Amount of ICD	Gasification Temperature	High-Temperature Strength Test Temperature
5%	1100 °C	800 °C/900 °C/1000 °C/ 1100 °C
10%	25 °C/1000 °C/1100 °C/1200 °C	
15%	1100 °C	
20%	1100 °C	

## Data Availability

The original contributions presented in this study are included in the article. Further inquiries can be directed to the corresponding author.
